# Attention Deficit Hyperactivity Disorder: Is There an App for That? Suitability Assessment of Apps for Children and Young People With ADHD

**DOI:** 10.2196/mhealth.7371

**Published:** 2017-10-04

**Authors:** Lauren Powell, Jack Parker, Naomi Robertson, Valerie Harpin

**Affiliations:** ^1^ School of Health and Related Research University of Sheffield Sheffield United Kingdom; ^2^ Department of Psychology University of Sheffield Sheffield United Kingdom; ^3^ Ryegate Children's Centre Sheffield Children's NHS Foundation Trust Sheffield United Kingdom

**Keywords:** attention deficit disorder with hyperactivity, mobile applications, technology

## Abstract

**Background:**

Attention-deficit/hyperactivity disorder (ADHD) is a complex highly comorbid disorder, which can have a huge impact on those with ADHD, their family, and the community around them. ADHD is currently managed using pharmacological and nonpharmacological interventions. However, with advances in technology and an increase in the use of mobile apps, managing ADHD can be augmented using apps specifically designed for this population. However, little is known regarding the suitability and usability of currently available apps.

**Objective:**

The aim of this study was to explore the suitability of the top 10 listed apps for children and young people with ADHD and clinicians who work with them. It is hypothesized that mobile apps designed for this population could be more suitably designed for this population.

**Methods:**

The top 10 listed apps that are specifically targeted toward children and young people with ADHD in the United Kingdom were identified via the Google Play (n=5) and iTunes store (n=5). Interviews were then undertaken with 5 clinicians who specialize in treating this population and 5 children and young people with ADHD themselves, to explore their opinions of the 10 apps identified and what they believe the key components are for apps to be suitable for this population.

**Results:**

Five themes emerged from clinician and young people interviews: the accessibility of the technology, the importance of relating to apps, addressing ADHD symptoms and related difficulties, age appropriateness, and app interaction. Three additional themes emerged from the clinician interviews alone: monitoring symptoms, side effects and app effect on relationships, and the impact of common comorbid conditions. The characteristics of the apps did not appear to match well with the views of our sample.

**Conclusions:**

These findings suggest that the apps may not be suitable in meeting the complex needs associated with this condition. Further research is required to explore the value of apps for children and young people with ADHD and their families and, in particular, any positive role for apps in the management of ADHD in this age group. A systematic review on how technology can be used to engage this population and how it can be used to help them would be a useful way forward. This could be the platform to begin exploring the use of apps further.

## Introduction

### ADHD, Technology, and Mobile Apps

Attention-deficit/hyperactivity disorder (ADHD) is a neurodevelopmental disorder characterized by three core symptoms: inattention, impulsivity, and hyperactivity, which can have a profound impact on the individual, their family, and their community [1-3]. It is a highly comorbid [4-6] chronic disorder and has a prevalence of 3% to 5% in school-age children worldwide [7]. Furthermore, 80% to 85% of these children will continue to be impaired by their ADHD symptoms as adolescents and 60% as adults [2,8-16]. Indeed, the presence of ADHD increases the risk of premature death [17]. Those whose ADHD persists into adulthood are more likely to engage in criminality and substance abuse [11,18,19]. Globally, ADHD management involves a combination of nonpharmacological and pharmacological interventions [20-22]. In mild to moderate cases, behavioral interventions such as psychoeducation and cognitive behavioral therapy are used alone, whereas in more severe cases, it is recommended that both pharmacological and nonpharmacological approaches are used concurrently [20,21]. Currently, the resources available to the service provider may limit interventions. Therefore, interventions that can offer support with minimal input from the clinic or school and can be generalizable would be highly desirable. Contemporary forms of engaging children and young people, such as the use of technology, could have potential in facilitating greater self-awareness, improving self-management skills or management for carers, and managing the condition into adulthood [23].

Xu et al reviewed 19 studies assessing technology use in students with ADHD in the age group of 4 to 19 years. The authors concluded that as yet there is very little evidence to support the effectiveness of such interventions [24]. Another recent review of what the authors describe as the most representative studies of the past decade assessed the findings of research that investigated the use of varying technologies with young people diagnosed with ADHD [25]. Studies included involved the use of a handheld device to help organize daily activities [26] or self-monitor symptoms [27], software to improve reading speed [28], and games to improve mathematical ability [29]. The success of these technologies was measured in a number of ways, including observational data [26-29], the Behavioral Assessment of Dysexecutive syndrome [26], qualitative interviews [26], reading speed, and time to complete assignments [28]. The authors concluded that they believe technology can enhance the learning of people with attention difficulties; however, the evidence base for this remains limited.

Another study looked at a computer mission game that aims to promote behavioral learning and organization of daily skills such as time management and planning or organizing [30]. Children played the game either 3 or 8 times a fortnight. A user satisfaction survey showed that between-group differences for game satisfaction were not observed, but children did enjoy the game and reported learning from it [30].

Technology has also been used by clinicians to aid diagnosis of ADHD and to monitor outcomes. For example, the quantified behavior (Qb) Test uses the Continuous Performance Test to produce a visual graph of the three core ADHD symptoms [31] and has been used to assist with the diagnosis of ADHD [32]. The primary outcomes of this study are time to diagnosis and diagnosis accuracy. The secondary outcome measures are clinician’s diagnostic confidence and routine clinical outcome measures. The authors are also conducting a qualitative assessment of the feasibility and acceptability of incorporating the QbTest into routine practice. A Web-based technology, *Health Tracker*, has also been used for parents, children, and professionals to track the long-term outcomes of children and young people with ADHD to enable more effective treatments and a more efficient service delivery [33].

The use of mobile phones and mobile devices has risen dramatically over recent years. Indeed, a UK report cofunded by the European Union found that 93% of children and adolescents aged 9 to 16 years in the United Kingdom access the Internet at least weekly and over half of these do so via mobile devices such as mobile phones [34]. As a result, mobile apps are also increasingly popular, and there has been some success in using them to engage children or young people with ADHD. These apps can include games, information about ADHD diagnosis and ADHD treatment, various ADHD tests, task managements, and reminders [35]. These attempts often incorporate reward [36-38], bright colors, and varied visual stimuli [37,39-43]. Examples include using apps said to monitor behavior in ADHD [41], improve behavior [44], improve organization skills [39,43] address medication compliance [36,45], improve reading motivation and summarization [40], and improve cognition through the use of games [37].

Currently available research evaluating apps for children and young people with ADHD comprises single case studies [37], technology development reports [36,42], and small sample sizes [36,37,40,42-44]. The conclusions drawn by these evaluation attempts of these apps include benefits for children and young people with ADHD, such as the app can improve organization and time management, reduce conflicts with parents during morning routines [43], and improve academic improvement [37] and *ontask* behaviors [44]. These claims are based on small sample sizes of 2, 1, and 8, respectively. Each evaluation method varied, and there was little consistency in the way apps were assessed.

Although an increasing number of apps are being promoted for use by or with [46] individuals with ADHD, there is still little guidance to support the reliability, validity [35], and suitability of currently available apps. There are few rules and regulations around what apps are suitable and for whom. In England, a review of the National Health Service (NHS) Choices apps library was principally focused on three components: (1) compliance with the Data Protection Act, (2) evidence of efficacy, and (3) relevance to British individuals. Many identified apps did not have an evidence base, and it was shown that privacy and data security was not suitable [46]. Within a week, the library was gone.

The concept of the use of apps to manage ADHD in children and young people is in its infancy. The research base is thin and is hugely outweighed by the number of apps available, thus suggesting that available apps are often not evidenced-based [35] and may not be suitable for their complex target population. Therefore, this study aims to identify and evaluate currently available apps that are aimed at this population by gaining the opinions of children and young people with ADHD and specialist clinicians. These opinions will be used to ascertain what they believe would make an app suitable for this population. It is hypothesized that the selected apps will not be suitable for this population, as apps generally have a thin or no evidence base, and this complex population has very specific needs.

### Methodology

This research involved initially the identification of the top 10 listed apps aimed at children and young people diagnosed with ADHD. The apps were identified as top 10, which was deemed as a reasonable and manageable figure [47,48] as there is a limited number of apps a consumer will search for. The authors believe that the top 10 apps will be viewed as the best 10 apps as they are the top 10 apps for a reason. Subsequently, young people with ADHD tested the apps, and they were then interviewed to ascertain their views on the apps and to explore what they believed the key components are for apps to be helpful for them. Clinicians were also interviewed to explore their insights into how to make apps successful for this population.

### Research Question

Are the top 10 listed apps specifically designed and marketed for children and young people with ADHD suitable, and what are the key components for apps to be suitable for this population?

## Methods

### Search and Identification of Mobile Apps

In June 2016, a search of mobile apps in the Apple iTunes store and the Android Google Play store in the United Kingdom was conducted. iTunes and Google Play were chosen, as they are the two largest and most popular app stores [49]. Apps for iPads and tablets were reviewed rather than phones, as apps are available on phone and tablet devices. For this study, apps available on tablets have been reviewed as they are easier to discuss with children, young people, and clinicians. These databases were selected as they display systematically organized app rankings defined by algorithms unique to each app store, commonly known as app store optimization (ASO). For Apple, the primary factor is the number of downloads; however, there are also many other secondary factors such as keywords and visuals [50]. Similarly, the Android database is filtered according to multiple criteria, including the volume of ratings, value of ratings, and download growth [51]. Although this gives rise to potential bias, as apps are selected according to the database’s own ASO, it is to some extent unavoidable unless all of the search results are downloaded for testing [48]. Where the authors do acknowledge that other app stores such as Amazon, Windows, and Blackberry do exist, currently, these do not have enough of a market share to be considered.

The search term we used was “ADHD.” This is because other search terms such as “ADD,” “children,” and “Young people” did not provide different results to the “ADHD” searches. The term “ADHD” was searched in both of the listed app stores.

Preliminary screening was conducted based on app titles, full marketing description, and screenshots of the apps potentially relevant for inclusion. The first five apps that fit the inclusion criteria (Textbox 1) were included from each app store, giving a total of 10 apps for inclusion.

**Figure 1:**
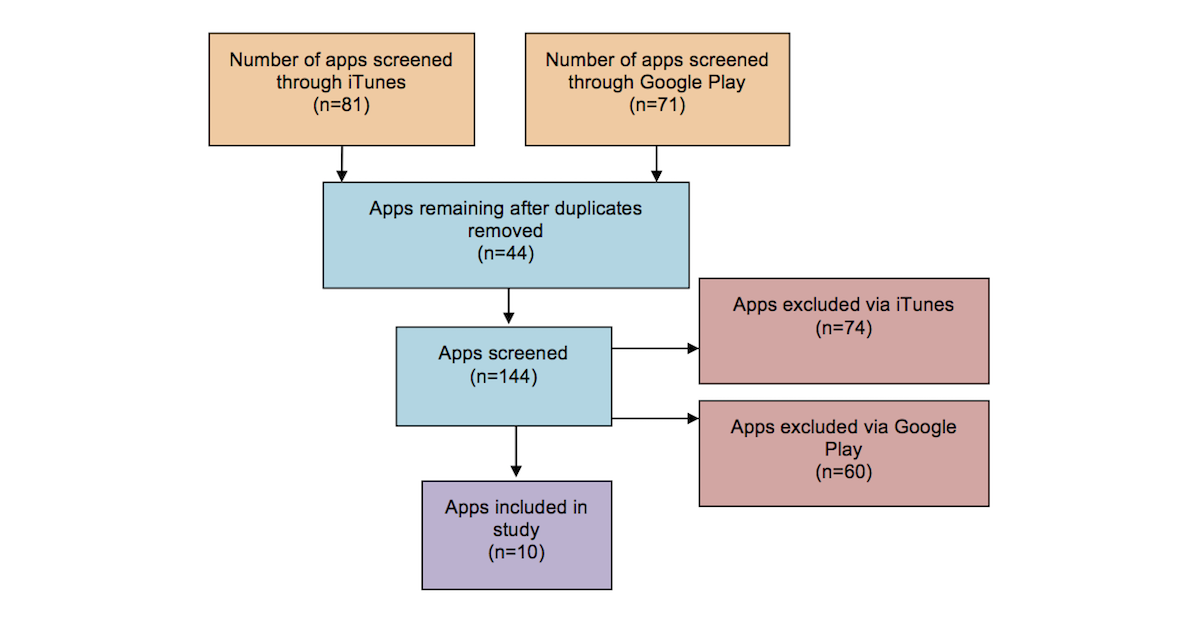
App selection process. The number of apps screened is the number of apps that had to be screened before identifying five apps for each database that fit the inclusion criteria. Eight apps were duplicated across both databases; therefore 144 apps were screened before the final ten were selected.

Duplicate apps were then removed (see Figure 1). This was applicable if there was more than one version of an app. It was decided that the app version to be included was the app that appeared first on the app store list.

The five apps from iTunes that fit the inclusion criteria were downloaded onto an Apple iPad mini (model: A1489), and the five apps from Google Play were downloaded to a Samsung device (model: GT-P5220). The app contents were summarized by 2 members of the team (LP and NR). The apps were simply summarized into a tabular format to help assist clinician participants during the semistructured interviews (see Multimedia Appendix 1). Tables 1 and 2 also give a brief overview of the apps claims. Multimedia Appendix 1 was given to clinicians during interviews for their information.

**Inclusion and exclusion criteria of apps.**

Inclusion criteria

- States aimed at attention-deficit/hyperactivity disorder (ADHD)
- The user is a child or young person with ADHD or attention deficit disorder (ADD)
- Mobile app
- App is available in the English language

Exclusion criteria

- Does not state that app is aimed at ADHD or ADD
- Not targeted at the child or young person with ADHD or ADD (eg, targeted at parents or clinicians only)
- Not a mobile app
- Not available in the English language
- Duplicate app

### Participants and Recruitment

A convenience sample of 5 clinicians and 5 children and young people were invited to take part. Children and young people were recruited via a Family Action group (a national charity that provides support for families) and a Web-based ADHD community group. Clinicians were recruited via the NHS (Child and Adolescent Mental Health Service and a Pediatric Neurodisability Service in the South Yorkshire region). Four clinicians were medical staff and one a specialist nurse. Participants were recruited until data [52] were achieved. Eligibility criteria for clinicians were that they had to be employed by a service that treats children and young people with ADHD and to specialize in treating this population themselves. Eligibility criteria for the children and young people were (1) must have a confirmed ADHD diagnosis and (2) must be in the age group of 6 to 17 years.

### Procedure

The study received ethical approval from the University of Sheffield’s School of Health and Related Research Ethics Committee (references 007880, 010768).

Clinicians were approached via email. A University of Sheffield researcher visited a group for parents of children with ADHD. Parents are referred to this group by NHS clinicians when their child receives a diagnosis of ADHD. The researcher explained the study to the parents and the parents discussed it with their children at home. Age-appropriate information sheets were also provided for the parents to give to their children. If their child wanted to take part in the study, the parent either contacted the researcher on their behalf or, with permission the researcher, contacted the parent to discuss and arrange an interview appointment. Ten separate semistructured interviews took place in the Sheffield region and were held in locations convenient to the participants (either NHS clinic for clinicians or home for the young people) from June to July 2016 for the clinicians and from October to November 2016 for the young people. Interviews lasted up to 60 min with the clinicians and up to 45 minutes with the child or young people.

Before the interviews, the clinicians provided written informed consent. Children and young people provided written assent and their parents consented on their behalf.

Information about the study was sent to all participants at least 1 week before interviews took place. At the beginning of each interview, the study was explained to the participants and questions were answered.

Clinicians were presented with all 10 apps identified. As two of the 10 apps were aimed at a younger age group (3-7 years) and all children or young people interviewed were over the age of 7 years, they were given eight apps to review. Participants were given the opportunity to use the apps themselves during the interview. Interview discussions occurred after participants examined each app. These discussions were guided by an interview schedule covering their views in four key areas:

- What makes a successful app?
- What doesn’t make a successful app?
- How could an app function benefit this population
- How could apps help manage ADHD or address difficulties in young people

The two groups provided two unique perspectives: a user perspective and a clinical perspective. Participants also completed a short questionnaire on their demographic characteristics.

### Data Analysis

All interviews were audio-recorded and transcribed verbatim. Thematic analysis [53] was used to search for data patterns within and across the participant groups. LP and JP independently identified codes and themes from the transcripts. Discrepancies were resolved through group discussion in an iterative fashion between authors. Themes identified aimed to capture the essence of the participants’ views.

Additionally, participants identified characteristics they believe apps should include if they are to be suitable for this population. These characteristics are presented in Table 3 and are accounted for within the qualitative analysis. Authors tabulated these characteristics and assessed each of the 10 apps. They documented how many of these characteristics identified by participants were present in the apps assessed in this study.

## Results

### ADHD: Is There an App for That?

Five apps were identified from Google Play and five from iTunes in July 2016. Tables 1 and 2 describe the claims the apps make within their individual descriptions and their contents. Table 3 demonstrates the app characteristics identified by participants during semistructured interviews and which apps possessed these characteristics. These characteristics are accounted for within the themes that emerged from the qualitative analysis and were discussed and agreed by participants.

**Table 1:** Characteristics of the apps downloaded from iTunes.

App	App claims	How apps meet their claims
1	Improves attention, concentration, focus, perceptual reasoning, academic performance, and inhibition impairments	Games to improve cognition
2	Improves self-control, reduces hyperactivity, improves attention, concentration, and focus	Mindfulness training
3	Improves self-control, reduces hyperactivity, and improves attention, concentration, and focus	Mindfulness training
4	Improves attention, concentration, and focus	Different version of same game involves responding to stimuli as quickly as possible.
5	Improves academic performance	Games and lessons (for user to watch)

**Table 2:** Characteristics of the apps downloaded from Google Play apps.

App	App claims	How apps meet their claims
6	Addresses memory and provides information about ADHD^a^	Memory games, different levels, ADHD key concepts quiz. Dialogues with a cartoon character, links provided with ADHD information
7	Improves attention, concentration, and focus, and addresses memory	Three games (find all objects, find numbers, reaction times), 4 memory games
8	Visualize time moving	On-screen moving timer
9	Improves academic performance	Improve reading speed: different ways of presenting text, books for different reading abilities, comprehension quiz on text previously read
10	Provides motivation	Talking fitness avatar, games involving physical activity

^a^ADHD: attention-deficit/hyperactivity disorder.

**Table 3:** Summary of what makes an app suitable for a child or young person with attention-deficit/hyperactivity disorder (ADHD; left column), according to the children and young people with ADHD and the clinicians interviewed in this study and which apps identified in this study include these characteristics. Authors have examined each app against the criteria identified by participants and scored the apps out of 8 to highlight how they, mostly, are not in line with the needs of their target audience.

Characteristics identified by participants as likely to be positive	iTunes					Google Play				
	App 1	App 2	App 3	App 4	App 5	App 6	App 7	App 8	App 9	App 10
Visually pleasing (ie, includes bright colors)	Yes	Yes	No	Yes	No	Yes	No	Yes	No	Yes
Allows personalization so user can relate to app	No	No	No	No	No	No	No	No	No	Yes, change avatar’s clothes
Plays music	Yes	Yes, relaxation	Yes, relaxation	No	Briefly in some sections, not all	Yes	No	No	No	No
Provides audio feedback (ie, makes a sound when user interacts with app)	Yes	No	No	Yes, pig snorts at a correct response	Only in some sections	Yes	No	No	No	Yes
Involves instant reward	No	No	No	No	No	Yes, collect coins, different levels	No	No	No	Yes, user gains points
Is interactive	Yes, games	No	No	Yes	No	Yes	No	Yes	No	No
Involves symptom monitoring component or ADHD^a^-related monitoring component (eg, diet)	No	No	No	No	No	No	No	No	No	No
Involves component that encourages healthy relationships with others	No	No	No	No	No	No	No	No	No	No
App score (out of 8)	4	2	1	3	2	5	0	2	0	4

^a^ADHD: attention-deficit/hyperactivity disorder.

**Table 4:** Demographic characteristics of young people.

Unique ID	Age, in years	Gender	Length of ADHD^a^ diagnosis (years, months)	Other diagnoses	Current prescribed ADHD medication	Medicated during interview?
YP1^b^	10	Male	1, 11	Autism spectrum disorder (ASD)	Concerta XL, melatonin	Yes
YP2	13	Female	0, 7	ASD, anxiety	Equasym XL	No
YP3	9	Male	6, 5	ASD	None	No
YP4	8	Male	0, 5	Not applicable	Equasym XL, Methylphenidate Immediate release	Yes
YP5	8	Female	1, 0	ASD, generalized anxiety disorder, sensory processing difficulties	Methylphenidate Immediate release	No

^a^ADHD: attention-deficit/hyperactivity disorder.

^b^YP: young person.

### Participant Characteristics

Ten participants, recruited in 2016, were included in this study: 5 clinicians and 5 young people diagnosed with ADHD aged 8 to 13 years (2 female, 3 males). Participant characteristics are reported in Table 4 (young people) and Table 5 (clinicians). Four of the five young people provided their ADHD medication as additional confirmation of diagnosis. One participant was not medicated. Four out of the five young people were recruited via an ADHD parent group in Sheffield. Parents were only referred to this group if their child was diagnosed with ADHD.

**Figure 2:**
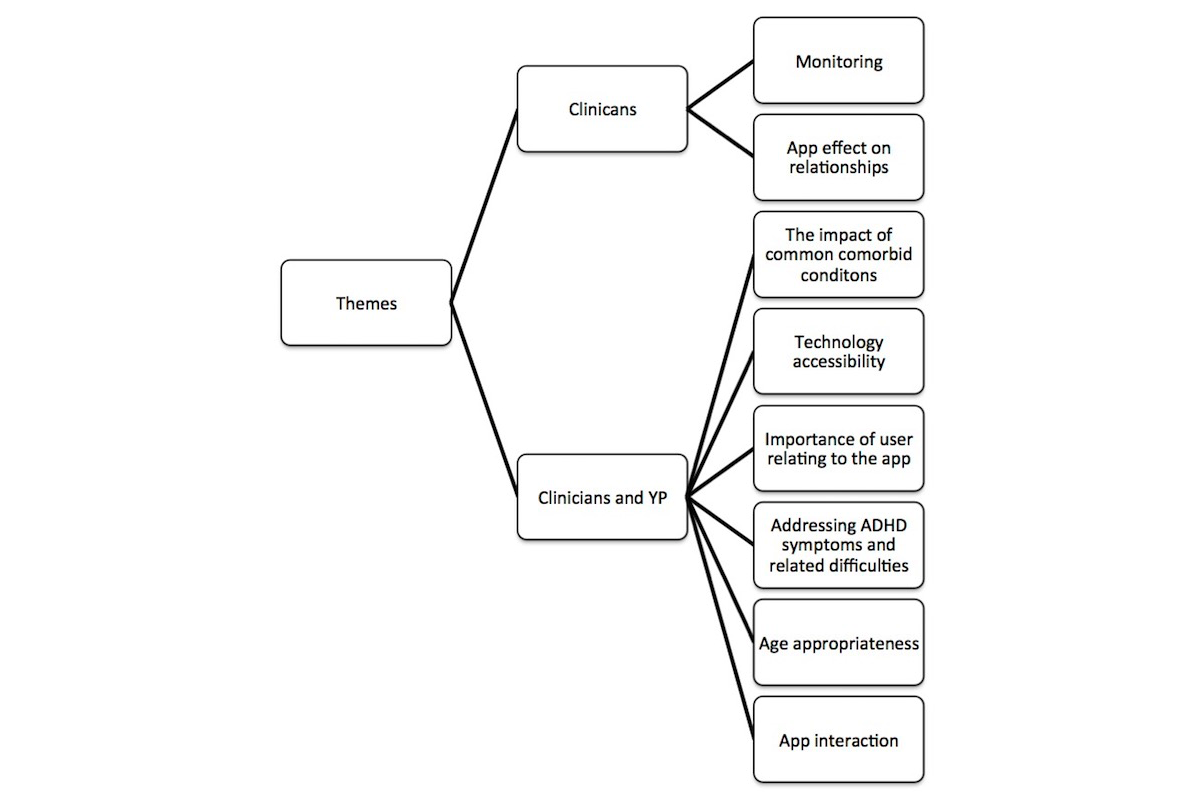
Summary of themes that emerged from the data and which participant perspectives they represent.

Data were collected until data saturation was reached. Transcripts were available for all 10 interviews. During analysis, agreement between the 2 primary coders was high. Seven themes were identified in total (see Figure 2). Where similarities between children or young people and clinician views emerged, from the data, they are combined and discussed under the same themes. These themes are identified, compared, and discussed below with illustrative quotations.

**Table 5:** Demographic characteristics of clinicians demonstrating a total of more than 57 years of experience working with children and young people with attention-deficit/hyperactivity disorder (ADHD).

Unique ID	Gender	Length of time working with population (years)	Current job title
HCP1^a^	Male	4	Specialist registrar in Child and Adolescent Mental Health Service
HCP2	Female	11	Consultant child and adolescent psychiatrist
HCP3	Female	22	Community mental health nurse
HCP4	Female	15	Associate specialist in pediatric neurodisability
HCP5	Female	5	Consultant child and adolescent psychiatrist

^a^HCP: health care professional.

Eight themes emerged from the data and are presented below with supporting quotations. They cover views specifically expressed by clinicians, which include the following: that apps could be used to monitor symptoms of children and young people with ADHD, apps could have both a positive and a negative effect on the relationships of child or young person with ADHD with others, and the impact of common comorbid conditions. Other themes expressed the views of both the clinicians and the children or young people. These themes involve discussion around accessibility issues of the technology, how important it is that apps consider ADHD symptoms and related difficulties, they should be age appropriate, preferably have an element that can be personalized so that the user can relate to the app, and similarly, the user should be able to interact with the app rather than simply watching a video clip or listening to audio recordings.

### Technology Accessibility

Young people (n=2) and clinicians (n=5) noted that the apps were not always reliable, as they often did not work properly or the log-in functions failed. This was a barrier when trying to engage them with the technology. It was recognized as “silly” (YP2) that the apps were all only available via iTunes or Google Play, which was problematic for 2 participants, as they did not have access to both. The young people also felt that it was a barrier to pay for apps, as they are not old enough to pay for them online (n=2). One participant noted that iPads could be slow and not load quickly enough, which was considered as “boring” (YP2). One participant noted:

Some didn’t register log-ins but that’s just the people that made it messing up and that.

YP1

Another participated stated:

...you have to like buy them but that’s annoying cos they should be free...I haven’t even got a credit card.

YP2

Clinicians were often skeptical of the claims the app developers made (n=3) and believed they lacked underpinning evidence (n=3), as noted below:

...it’s difficult through an app to build a child’s kind of confidence.

HCP1

So they are trying to just say that going on an app makes you less impulsive and reduces your hyperactivity which I very very much doubt.

HCP4

...quite bold non evidenced based statements aren’t they.

HCP

### Importance of User Relating to the App

All 5 young people noted a number of points relating to their wish to relate to the app; audio was important to all participants. Two young people described one of the voices on the app as a “robot” (YP1 and YP3) and as having a “creepy voice” that goes “on and on and on” (YP1); they said they wanted the voice to have “more expression” (YP1). Young people and clinicians noted that the apps should be visually attractive (n=10) and “colorful” (YP2, YP3, and YP4) or “pretty” (YP5). They liked the idea of changing some characters in the apps to represent themselves (n=5) or somebody else such as Justin Bieber, their favorite pop icon (YP2). Similarly, many of the young people believed that the character in apps is important (n=2):

...cos if there were no sound you’d just play something with no sound and it’ll be real boring.

YP3

...they (app developers) could change the voice a bit to like a character in like a like someone in a film.

YP4

Well his background needs changing to little boys bedroom...

YP1

...the trousers you could actually have trackies on like mine.

YP4

Similarly, the clinicians thought that the apps should be fun (n=3), visually attractive (n=5), use language (n=3) and accents (n=1) the child can relate to, and incorporate reward (n=1). It was discussed that relating to an app may not always be a positive experience for young people. One clinician stated that one of the apps appeared to be mimicking school, which a child may negatively relate to. Another clinician stated that a child might respond to a character on an app telling them what to do as opposed to a parent or school teacher:

...cos it just feels like being at school...and I think they’ve got to be fun.

HCP3

...the logo is very important...cos that’s what teenagers will see...people with ADHD aren’t going to read all the details they’re going to be very impulsive aren’t they and think oh that looks interesting and bang (download the app).

HCP4

### Addressing Attention-Deficit/Hyperactivity Disorder (ADHD) Symptoms and Related Difficulties

Young people noted a number of issues that involve addressing their ADHD symptoms. For example, 2 participants liked the idea that apps could be used to relax them (ie, assist with their hyperactivity).

Participants noted that gaining an instant reward such as “leveling up” or gaining coins during a game made them feel “happy” (YP2). One participant found apps had the opposite effect when she was denied rewards, such as leveling up (YP5). Four participants said it is important that an app contains lots of variety to keep YPs attention. One participant stated:

...it [the app] should make it so every time you complete one [game level] you get little coins and you can buy backgrounds and that.

YP1

Another participant noted:

It makes you focus on the pattern so you relax.

YP2

Similarly, clinicians also believed that reward and variety in apps are important to engage the user. One clinician believed that apps for ADHD have the ability to help improve their memory and inhibition impairments, thus targeting the impulsivity in ADHD. It was noted that the apps themselves could be a distraction to other important tasks (n=1):

...if they’re rewarded for each activity...they’re going to engage...cos they’re gonna get their dopamine hit each time.

HCP4

### Age Appropriateness

Young people (n=4) and clinicians (n=5) believed that it is important for the apps to be age appropriate for the user. Some participants said that some apps were a bit “babyish,” and some noted that one of the apps was a bit too old for a child because of the language used. One participant noted that one of the apps was describing a scene that was “hard to imagine” (YP1). The participants noted:

A bit childish, does it say for 13 [years old]?

YP2

...I’d rather have it when I’m older.

YP4

...it doesn’t feel as if it’s aimed at kids.

HCP3

I think it’s very immature, I dunno what the age range is but it feels like the cartoons I used to watch when I was little *laughs*.

HCP5

### App Interaction

Young people stated that apps must be interactive (n=3). They were less impressed and, at times, frustrated with apps that involved no interaction but simply listening or watching (n=3). They wanted to be involved and interact with the app itself, as noted below:

...how I didn’t do anything! This app is fake! Its fake! [the app said he had achieved the next level and he hadn’t done anything].

YP3

I didn’t like it seriously, it’s mind controlling! [as above, the app said she had achieved the next level and he hadn’t done anything].

YP5

I would like change it a bit so you can like actually do something cos and the moment on the writing you just you don’t...do anything...

YP4

One clinician also noted that the apps should incorporate learning and make it fun, have a catchy title and app logo, and a simple description. One person highlighted that ADHD medication could affect a child’s ability to engage with an app:

Some children who we see with ADHD will be medicated and some won’t and that will affect their levels of concentration...

HCP2

The following three themes were considered to be important by clinicians but not mirrored by the young people:

#### Monitoring

Clinicians suggested that apps could be used to monitor diet (n=2), a “mood component” (C1; n=2), and ADHD symptoms within this population. Some believed they would be useful for keeping a diary (n=3) of these, and one person believed apps would be useful to act as ADHD medication reminders:

A child and parent app then the parent can then monitor [ADHD symptoms].

HCP1

I think something about a diary in terms of perhaps providing prompts or things that they’ve got going on that day...

HCP2

I think it’s really good to encourage kids with ADHD to monitor the way they feel.

HCP3

#### App Effect on Relationships

One clinician was mindful that the use of apps could prompt “disharmony” in the home, causing conflict in terms of sharing with siblings (C2). They were also mindful that a number of the apps encourage the user to access them daily. This clinician believed that this could encourage a parent to nag their child to use the app, causing tension in the home as well:

...it kind of might at some point cause tension and then, you know disharmony at home and arguments and things like that, prompting them to try and get them to do.

HCP2

Others believed that as one common difficulty in ADHD is maintaining relationships with friends and family, apps could help facilitate these relationships rather than be harmful to them (n=2):

...err I think relationships with peers would be useful...

HCP2

I think peer relationships would definitely be one thing...if you’re looking at relationships you could also mention family relationships as well.

HCP1

#### The Impact of Common Comorbid Conditions

Additionally, they highlighted that ADHD is a highly comorbid condition and the apps don’t always account for this (n=2). One clinician shared that children with ADHD may have learning difficulties and be behind academically and that some of the apps used appeared challenging for the target age even without the young people having additional needs. One participant stated that autism spectrum disorders are a common ADHD comorbidity and that a symptom of ASD can be the inability to cope with multiple stimuli at once. For example, some of the apps were very visually “busy” with lots of background noise. This clinician described this as “sensory overload” (HCP4); something these users may not always be able to cope with:

...a lot of children as you know with ADHD have got autism as well and so sometimes things that are completely too noisy, too bright, unless they pick them, can be a bit of a problem.

HCP4

## Discussion

### Principal Findings

This study identified 10 apps that stated they were aimed at children and/or young people with ADHD. Five clinicians working with this population and 5 young people with ADHD were interviewed. Interviews involved sharing the 10 identified apps with participants and asking what they think would make an app suitable for children and young people with ADHD. Young people stated that technology unreliability can be a barrier when trying to engage them with the technology; young people and clinicians believe that the young people want to be able to relate to an app, clinicians wanted apps to target the ADHD symptoms of young people, young people and clinicians wanted apps to provide rewards, and young people wanted to be able to interact with an app. Additionally, 2 clinicians felt apps would be useful to monitor ADHD symptoms, diet, and mood and to improve family and peer relationships.

### Conclusions

ADHD is a chronic condition with around 60% going on to have some symptoms in adult life [2]. Treatment with medication for ADHD falls significantly during the second decade of life [54]—a time when many young people with ADHD struggle with school, family, and peer relationships and risks such as drug misuse. As with all chronic conditions, empowering patients to manage their own symptoms is crucial. Multimodal treatment is recommended for ADHD management [20], but in most countries, resources for ongoing nonpharmacological support are often thin.

Individuals with ADHD, and especially those with comorbid ASD, often enjoy technology and indeed are skilled with its use. Many apps are advertised for individuals with ADHD and their families [36,37,40,42-44], but little is known about their use and value. In this small study, we aimed to collect the views of the target group, children and young people with ADHD, and of clinicians who specialize in management of ADHD on what qualities they would like to see in an app and for ideas on when apps could be useful. The young people and clinicians whose views were sought in this study did not find that the apps reviewed fully met their expectations. The highest match was 5 out of 8; two apps scored 0 and the mean was 2.3 out of 8. Young people wanted to enjoy the technology and to use it for some specific tasks. The clinicians were keen to explore apps as a way to engage young people, motivate them to manage their difficulties, and target specific needs for particular young people.

The perspectives of both the clinicians and the young people are needed to build up a full picture. It is important to have the views of clinicians as to whether the apps promote positive behaviors and target specific needs. The views of the young people give guidance as to whether they would actually use the app and whether they found it enjoyable and/or helpful. Individuals with ADHD show most reliable responses to frequent positive reward, so this seems to be a prerequisite of a successful app for the young people themselves.

Both clinicians and their patients wanted apps to be technically reliable, to relate in some way to the user, to be age appropriate, and to be interactive. Both were also keen to exploit technology to help them, including, for example, an app to address ADHD symptoms in some way. App developers may advertise their apps to offer what clinicians and young people want, but as yet, there does not seem to be a real evidence base to help families and clinicians decide whether or not an app is likely to work for them. Claims that apps can improve ADHD symptoms need further exploring. Clinical networks and children, young people, and families could work with app developers to trial existing apps and develop new ones.

Clinicians also saw the use of apps as a way of collecting data from their patients. For example, some young people do not gain weight on ADHD medications [55]. An app could perhaps be used to remind a young person to eat and to record what, if anything, they do eat. If a young person is suffering from low mood, perhaps an app could be used to record this and share mood scores with the clinician. It can be extremely difficult for a young person with ADHD to reliably report their dietary intake or their mood over time when they attend appointments [56].

The marketplace for app development and specifically app development in health care continues to expand. It is easy to see that conflict could exist between the desire to make money from an app by producing it in a less-than-ideal way and marketing it quickly and spending time and money researching the benefits of an app. Indeed, once an app has been purchased, the developer may move on to the next project, and families and patients who purchase cheap apps are unlikely to complain if they do not work well for them.

There is a need for guidelines and standards for app developers, including a requirement for transparent trials of the usefulness of the app. Clearly, there is however, a balance between making these so time consuming and expensive as to deter developers.

To investigate the factors influencing technology acceptance, a model named the technology acceptance model (TAM) provides a basis for attitude measures with two technology acceptance variables: perceived usefulness (PU) and perceived ease of use (PEU) [57]. PU refers to “the degree to which a person believes that using a particular system would enhance his or her job performance.” PEU is defined as “the degree to which a person believes that using a particular system would be free from effort.” Research has shown that TAM has been one of the most influential models in explaining user acceptance of information technology (IT), and it has gained wide attention in the IT literature because it includes the psychological interaction of a user with technology (unpublished data, 2009 [58]). According to TAM, if users perceive a technology as useful and easy to use, they develop positive attitudes toward the technology.

Future research is needed on the value of apps for children and young people with ADHD and their families and, in particular, any positive role for apps in the management of ADHD in this age group. A systematic review on how technology can be used to engage this population and how it can be used to help them would be a useful way forward. This could be the platform to begin exploring the use of apps further.

## Appendix

Multimedia Appendix 1 Information about apps in this research.

## Abbreviations

ADD: attention deficit disorder
ADHD: attention-deficit/hyperactivity disorder
ASO: app store optimization
NHS: National Health Service
NIHR: National Institute for Health Research
PEU: perceived ease of use
PU: perceived usefulness
QbTest: quantified behavior test
TAM: technology acceptance model
