# Supplementary material for: Attention Deficit Hyperactivity Disorder: Is There an App for That? Suitability Assessment of Apps for Children and Young People With ADHD
Source: JMIR Mhealth Uhealth. 2017 Oct 4;5(10):e145. doi: 10.2196/mhealth.7371 (PMC5647456; doi:10.2196/mhealth.7371)
Supplement: Multimedia Appendix 1 [file mhealth_v5i10e145_app1.pdf]

Name of App	Price	Audience	App Claims	App features to meet app claim	Condition focus	Operating system
App 1	£3.99	YP with ADHD 4-12 years	Improves attention, concentration, focus, perceptual reasoning, inhibition impairments and academic performance.	A series of daily cognitive training games.	ADHD	iOS
App 2	£4.99	YP with ADHD and YP who wish to improve confidence, self control, calmness and concentration	Improves self-control, attention, concentration, ability to focus, confidence and increases calmness and reduces hyperactivity.	Guided mindfulness – audio relaxation recordings	ADHD, other children	iOS
App 3	£2.29	Parents, children/YP with ADHD	Improves self control, attention, concentration, ability to focus and reduces hyperactivity	Relaxation recordings	ADD, ADHD	iOS
App 4	£0.79	Children/YP with ADHD, other children wanting to improve attention, focus and concentration skills	Improves attention, concentration and ability to focus.	Alternating between varying game types all involving responding to on screen stimuli.	ADHD, other children	iOS
App 5	FREE	Children/YP with ADHD, LDs, those below reading age wishing to improve	Improves academic performance.	Interactive games and simple lessons.	ADHD, LDs	iOS
App 6	FREE	Children/adolescents with/without ADHD, parents, psychologists, drs, teachers, friends	Addresses memory, provides information about ADHD.	Test memory in some game levels, dialogue between cartoon characters, links provided to ADHD information.	ADHD	Android
App 7	FREE	ADHD 4-7 years, other children wanting to improve memory and attention.	Improves attention, concentration and ability to focus.	3 mini games; object finding, number searching and game involving reaction times.	ADHD, other children	Android
App 8	FREE	Children/YP with ADHD	Visualise time moving	Timer moving on screen	ADHD	Android
App 9	FREE (£1.70 full version)	Children/YP with ADHD or dyslexia 6-15years, other children wishing to improve reading ability	Improves academic performance.	Improve reading speed via varying types of training, presents text in different ways, comprehension quiz.	ADHD, dyslexia, other children	Android
App 10	FREE	ADHD, Autism, children with cognitive disabilities	Motivation.	Includes talking fitness avatar and games involving [illegible]	ADHD, Autism, cognitive disabilities	Android
